# Constructing bilayer and volumetric atrial models at scale

**DOI:** 10.1098/rsfs.2023.0038

**Published:** 2023-12-15

**Authors:** Caroline H. Roney, Jose Alonso Solis Lemus, Carlos Lopez Barrera, Alexander Zolotarev, Onur Ulgen, Eric Kerfoot, Laura Bevis, Semhar Misghina, Caterina Vidal Horrach, Ovais A. Jaffery, Mahmoud Ehnesh, Cristobal Rodero, Dhani Dharmaprani, Gonzalo R. Ríos-Muñoz, Anand Ganesan, Wilson W. Good, Aurel Neic, Gernot Plank, Luuk H. G. A. Hopman, Marco J. W. Götte, Shohreh Honarbakhsh, Sanjiv M. Narayan, Edward Vigmond, Steven Niederer

**Affiliations:** ^1^ School of Engineering and Materials Science, Queen Mary University of London, London, UK; ^2^ School of Biomedical Engineering and Imaging Sciences, King’s College London, London, UK; ^3^ National Heart and Lung Institute, Imperial College London, London, UK; ^4^ Center for Research in Advanced Materials S.C (CIMAV), Chihuahua, Mexico; ^5^ College of Medicine and Public Health, Flinders University, Adelaide, Australia; ^6^ Bioengineering Department, Universidad Carlos III de Madrid, Madrid 28911, Spain; ^7^ Department of Cardiology, Gregorio Marañón Health Research Institute (IiSGM), Hospital General Universitario Gregorio Marañón, Madrid 28007, Spain; ^8^ Center for Biomedical Research in Cardiovascular Disease Network (CIBERCV), Madrid 28029, Spain; ^9^ R&D Algorithms, Acutus Medical, Carlsbad, CA, USA; ^10^ NumeriCor GmbH, Graz, Austria; ^11^ Gottfried Schatz Research Center-Biophysics, Medical University of Graz, Graz, Austria; ^12^ BioTechMed-Graz, Graz, Austria; ^13^ Department of Cardiology, Amsterdam UMC, Amsterdam, The Netherlands; ^14^ Electrophysiology Department, Barts Heart Centre, Barts Health NHS Trust, London, UK; ^15^ Department of Medicine and Cardiovascular Institute, Stanford University, Palo Alto, CA, USA; ^16^ IHU Liryc, Electrophysiology and Heart Modeling Institute, Fondation Bordeaux Université, Bordeaux, France; ^17^ IMB, UMR 5251, University Bordeaux, Talence 33400, France; ^18^ Turing Research and Innovation Cluster in Digital Twins (TRIC: DT), The Alan Turing Institute, London, UK

**Keywords:** cardiac arrhythmia, computational model, *in silico* trial, patient-specific cardiac model, digital twin

## Abstract

To enable large *in silico* trials and personalized model predictions on clinical timescales, it is imperative that models can be constructed quickly and reproducibly. First, we aimed to overcome the challenges of constructing cardiac models at scale through developing a robust, open-source pipeline for bilayer and volumetric atrial models. Second, we aimed to investigate the effects of fibres, fibrosis and model representation on fibrillatory dynamics. To construct bilayer and volumetric models, we extended our previously developed coordinate system to incorporate transmurality, atrial regions and fibres (rule-based or data driven diffusion tensor magnetic resonance imaging (MRI)). We created a cohort of 1000 biatrial bilayer and volumetric models derived from computed tomography (CT) data, as well as models from MRI, and electroanatomical mapping. Fibrillatory dynamics diverged between bilayer and volumetric simulations across the CT cohort (correlation coefficient for phase singularity maps: left atrial (LA) 0.27 ± 0.19, right atrial (RA) 0.41 ± 0.14). Adding fibrotic remodelling stabilized re-entries and reduced the impact of model type (LA: 0.52 ± 0.20, RA: 0.36 ± 0.18). The choice of fibre field has a small effect on paced activation data (less than 12 ms), but a larger effect on fibrillatory dynamics. Overall, we developed an open-source user-friendly pipeline for generating atrial models from imaging or electroanatomical mapping data enabling *in silico* clinical trials at scale (https://github.com/pcmlab/atrialmtk).

## 1. Introduction

Therapy approaches for irregular heart rhythms including atrial fibrillation (AF) are suboptimal, which is in part because these therapies are not personalized to the patient. Personalized computational models may be used to predict individual patient response to therapy [1], to predict patient outcomes, to guide individual therapy [2] or for *in silico* trials [3]. Atrial models may be constructed from a variety of different data types, including electroanatomical mapping (EAM) shells or medical imaging data (magnetic resonance imaging (MRI) or computed tomography (CT)), which are segmented to produce a personalized anatomy. The degree of model personalization and the number of cases included in a study typically depends on the study aims, data availability and applicability of tools and methodology. Typically model construction pipelines require significant time and expertise from trained users, significant computation time and may not be reproducible. As computational medicine moves towards both large *in silico* trials and personalized model prediction on clinical timescales, it is imperative that these limitations in model construction pipelines are overcome.

We previously presented a reproducible pipeline for constructing left atrial (LA) models [4] using a universal atrial coordinate (UAC) system [5] to map fibres to each model from an atlas. Recent clinical studies and mechanistic modelling studies [6,7] have highlighted the importance of the right atrium in understanding AF and personalizing treatment approaches, motivating the extension of atrial modelling to biatrial modelling. Approaches to constructing personalized models include Piersanti *et al.* [8] who developed a Laplace–Dirichlet rule-based method for assigning fibres to the atria and ventricles. A further approach is the AugmentA pipeline developed by Azzolin *et al.* [9] for generating atrial models with fibres. They demonstrated AugmentA on 29 LA datasets, and used a statistical shape model to generate a right atrial (RA) anatomy for each LA anatomy, with rule-based fibres.

Here, we build on these approaches to overcome the challenges of constructing biatrial models at scale through developing a robust, open-source pipeline. The pipeline incorporates atrial structures in the models, including crista terminalis, pectinate muscles (PMs), the sinoatrial node (SAN) and Bachmann’s bundle (BB). Atrial fibres are included from a choice of atlas fibre distributions, including rule-based and data driven diffusion tensor MRI datasets. Models are constructed as either a coupled surface (bilayer) or volumetric representations. These tools are provided open-source as a user-friendly workflow. We apply these methodologies to create a cohort of 1000 biatrial bilayer and volumetric models from CT-derived statistical shape model data, and then investigate the effects of model type (bilayer or volumetric mesh) on AF dynamics and ablation outcome. We also apply the methodologies to EAM and MRI data, and investigate the effects of the choice of fibre map on paced and arrhythmia activation patterns. We use these large simulation studies to investigate the effect of model choice on *in silico* trial and personalized model predictions, and to investigate the relative impact of model type, fibres and fibrosis on fibrillatory wavefront patterns.

## 2. Methods

**Figure 1.**
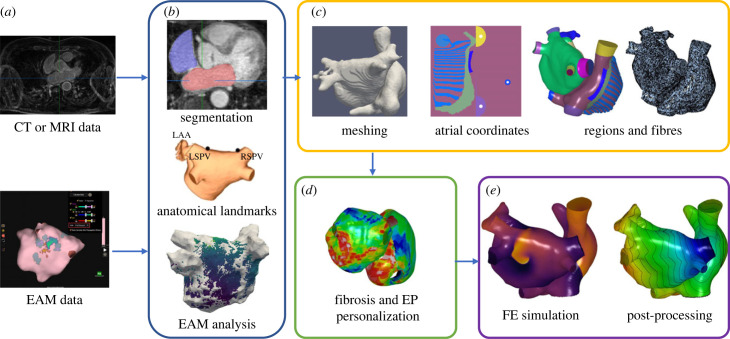
Methods schematic outlining the steps involved in patient-specific model construction. These include: (*a*) data inputs (imaging or electroanatomic mapping (EAM) data); (*b*) segmentation and anatomical landmark selection; (*c*) mesh generation with atrial coordinate calculation, regions and fibres; (*d*) model calibration to fibrosis or electrophysiology (EP) data and (*e*) finite-element (FE) simulations and post processing.

We first detail the types of data these methodologies could be applied to and the specific datasets used in this study in §2.1. We then describe the landmark selection (§2.2); and adaptions of our algorithms to cases that do not include pulmonary vein or appendage tissue (§2.3). We present scalar mapping methodologies for including atrial structures and interatrial connections in bilayer models (§2.4). We then describe techniques for constructing models as volumetric models (§2.5). We present vector mapping for including a range of fibre atlases in the models for bilayer representations (§2.6) and volumetric meshes (§2.7). We then describe methodologies for incorporating atrial fibrosis in models (§2.8). Finally, we detail our simulation set-up and post-processing techniques applied to compare arrhythmia dynamics (§2.9). Figure 1 provides an overview schematic with the key steps of the methodology.

### 2.1. Data sources

We demonstrated the pipeline on CT data, MRI data and EAM data as follows.

Anatomical LA and RA meshes were extracted from 1000 instances of a CT-derived statistical shape model, which are available to download on Zenodo (https://zenodo.org/record/4506930) [10]. These meshes are provided as unstructured tetrahedral meshes that were generated from four-chamber segmentations using the Computational Geometry Algorithm Library (CGAL) [11] with an average edge length of 1 mm. We post-processed these tetrahedral meshes by using meshtool software (https://bitbucket.org/aneic/meshtool) to extract the LA surfaces, RA surfaces and associated volumes from the four-chamber meshes [12]. We used the epicardial surfaces for constructing bilayer models.

Late-gadolinium enhancement MRI (LGE-MRI) and magnetic resonance angiograms (MRA) from Hopman *et al.* [7] were used for magnetic resonance mesh construction. The LA and RA blood pool were semi-automatically segmented from the MRA images using CemrgApp software [13] (http://cemrgapp.com/) to produce LA and RA endocardial surface meshes that were suitable for constructing bilayer models.

LA and RA surfaces were extracted from the EnsiteX EAM system (Abbott). For all data types, meshes were stored in vtk and stl formats.

### 2.2. Mesh pre-processing and landmark selection

For the MRI and EAM datasets, mesh pre-processing steps were applied to first open the mesh at the pulmonary veins, vena cava, coronary sinus, mitral valve and tricuspid valve. This was applied using a sphere clipping tool available in Paraview software (https://www.paraview.org/) [14].

To select atrial landmarks, a Python script written using the PyVista library [15] was used to enable the user to quickly click on landmark locations, and these locations were written to text files. For all meshes, general LA landmarks were selected as follows: right superior pulmonary vein (RSPV), right inferior pulmonary vein (RIPV), left inferior pulmonary vein (LIPV), left superior pulmonary vein (LSPV), the tip of the left atrial appendage (LAA) and the base of the LAA. Specific landmarks were selected as follows: (i) on the lateral wall, in line with the LSPV, posterior of the LAA; (ii) on the septal wall, in line with the RSPV (at the fossa ovalis); (iii) at the junction of the LA body and LSPV, at the centre of the posterior wall path; (iv) at the junction of the LA body and RSPV, at the centre of the posterior wall path.

Correspondingly, general RA landmarks were selected as follows: inferior vena cava (IVC) path choice, coronary sinus (CS), roof between superior vena cava (SVC) and IVC, SVC path choice, the tip of the right atrial appendage (RAA), the base of the RAA. Specific landmarks were selected as follows: (i) lowest point at the junction of the RA body and SVC; (ii) lowest point at the junction of the RA body and IVC; (iii) in line with the SVC, septal of the RAA; (iv) in line with the IVC; (v) at the junction of the RA body and SVC, at the level of the roof and (vi) at the junction of the RA body and IVC, at the level of the roof. All landmarks are illustrated on the github examples page.

For MRI and EAM meshes, atrial tissue for each of the pulmonary veins, appendages, vena cava and coronary sinus were automatically labelled using a series of Laplace–Dirichlet solves. For the left atrium, a Laplace–Dirichlet solve was computed using openCARP software [16,17] for each pulmonary vein, with boundary conditions corresponding to 1 along the chosen pulmonary vein boundary nodes, and 0 along the mitral valve boundary nodes. A manually chosen threshold was selected that approximated the ostia location and applied to assign all nodes with Laplace–Dirichlet solution greater than this threshold to a pulmonary vein region. The pulmonary vein regions were then assigned a specific pulmonary vein label depending on which landmark the region was closest to. For the appendage, a Laplace–Dirichlet solve was computed with 1 assigned at the LA appendage tip landmark, and 0 at the mitral valve. Again, a manually chosen threshold was applied to label appendage tissue as nodes with solution value greater than this threshold.

Similarly, for the right atrium, this approach was applied with boundary nodes at 1 for each of the SVC, IVC and CS in turn, and 0 at the tricuspid valve. These regions were then labelled using a threshold for the Laplace–Dirichlet solve following the same approach that was developed for the PV labelling. The RA appendage was labelled using the same methodology that was applied to the LAA.

Finally, clipped and labelled meshes were re-meshed to a standard resolution suitable for simulation studies using meshtool software (e.g. 0.3 mm average edge length, https://bitbucket.org/aneic/meshtool). Mesh labelling and re-meshing were automated across all cases through the use of bash scripts. Examples are provided to apply this pipeline.

### 2.3. Universal atrial coordinate calculation with or without pulmonary vein tissue

**Figure 2.**
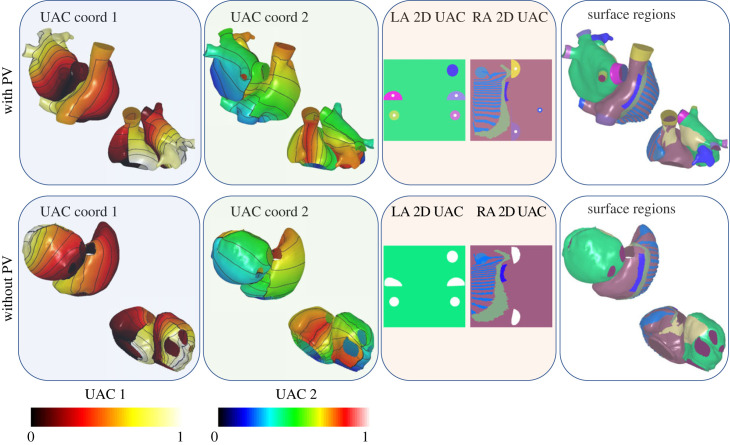
Two-dimensional universal atrial coordinates (UAC) for models with or without pulmonary vein tissue. Panels show UAC coordinate 1, UAC coordinate 2, 2D UAC representation of the LA and RA, and meshes with surface regions. The original set-up with pulmonary vein tissue is shown at the top, and the adapted methodology without pulmonary vein tissue is shown at the bottom. UAC 1 is displayed using the *hot* colour map and UAC 2 is displayed using the *jet* colour map, where both range from 0 to 1.

We extended the UAC system to work for cases in which the pulmonary veins, vena cava and appendages had been clipped and removed from the mesh; for example, in the CT statistical shape atlas dataset used in this study. Specifically, we removed boundary conditions previously included at the structure openings. The boundary conditions along the junction of the atrial body and PV, LAA and vena cava in the original UAC formulation were included equivalently at these boundary nodes in the clipped meshes since these boundaries represent the junctions between the atrial body and structures. This is shown in figure 2.

### 2.4. Universal atrial coordinate for atrial structure assignment and interatrial connections in bilayer meshes

Biatrial bilayer models were constructed from LA and RA epicardial or endocardial shells as follows. For MRI meshes, shells represented the LA or RA blood pool segmentation, and so were assigned as the endocardial surface. For CT-derived statistical shape models, epicardial surfaces were extracted from the volumetric meshes for constructing bilayer models to ensure LA and RA surfaces were close together. These endocardial (for MRI) and epicardial (for CT-derived statistical shape models) surfaces were then duplicated and projected 0.1 mm epicardially or endocardially, respectively, to create two layers. Equivalent nodes on the two LA surfaces were assigned the same UAC value and connected through line element connections. The projection distance used here is an arbitrary value because atrial wall thickness is included in the monodomain model simulations through the choice of line element coupling coefficients, following Labarthe *et al.* [18].

UACs were used to map atrial structures to the meshes, including crista terminalis, PM, BB and the SAN. These were included in an atlas bilayer mesh from Labarthe *et al.* [18] together with their UAC locations. For bilayer models, the mesh at the equivalent UAC locations in the new LA and RA shells was duplicated and projected endocardially or epicardially to form these regions. Nodes on these structures were connected to their equivalent nodes on the LA or RA epicardial surfaces through linear connections.

Interatrial connections were mapped to the meshes at BB, the CS and along the septal wall. At BB this was implemented through linear connections between a line of nodes on the LA and RA components of BB. For the CS, this was implemented through joining nodes on the half of the CS boundary closest to the LA epicardium to nodes on the LA epicardium using linear elements. Septal connections were implemented by joining LA epicardial nodes within a distance threshold of selected locations to their closest RA epicardial nodes using line connections.

### 2.5. Volumetric models: universal atrial coordinate extension

Volumetric UAC for each of the 1000 CT-derived statistical shape instances were constructed by three UAC solutions for each LA and RA volumetric tetrahedral mesh. The first two UAC coordinates were calculated for each of the LA and RA endocardial and epicardial triangulated surface meshes, as described above. Then these solutions were used as boundary conditions for the tetrahedral mesh Laplace–Dirichlet solve as follows. To calculate the first UAC coordinate on the LA volumetric mesh, the endo and epicardial LA surface mesh values for UAC coordinate 1 were assigned to the surface nodes of the volumetric mesh. A Laplace–Dirichlet solve was then computed to calculate UAC coordinate 1 through the LA volume. The same approach was used to calculate the second UAC coordinate on the LA volume, and then an equivalent approach calculated the first two UAC coordinates for the RA volume.

**Figure 3.**
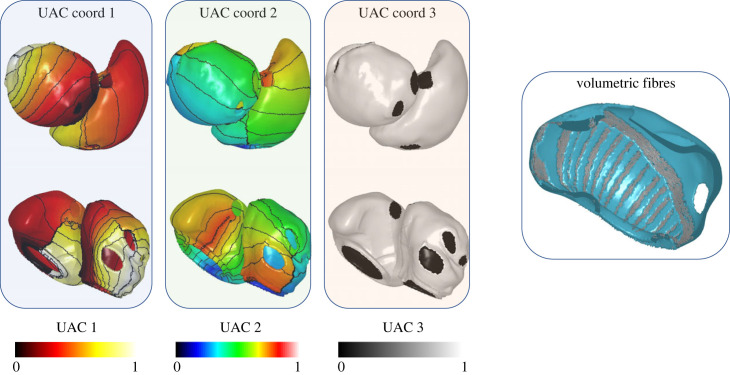
Volumetric atrial coordinates showing three atrial coordinates and transmural fibres. Volumetric fibres are displayed for the PM and crista terminalis regions of the model in the final panel. UAC 3 has the endocardium in black at a coordinate of 0, and the epicardium in white at a coordinate of 1.

For volumetric meshes, a third coordinate is required to provide a measure of transmurality. This was calculated using the following approach for each of the LA and RA volumetric meshes in turn. The endocardial nodes on the LA and RA volumetric meshes were assigned a value of 0, and the epicardial nodes on each mesh were assigned a value of 1. Laplace–Dirichlet solves were computed for the LA and RA volumetric meshes to calculate the third transmural coordinate. This is demonstrated in figure 3.

### 2.6. Including atrial fibres: bilayer models

Atrial fibres were mapped to surfaces from a choice of atlases using UAC to perform vector field mapping, as previously described [19]. Atlases were stored as endocardial fibre fields for each atria, and epicardial fibre fields for each atria. For the Labarthe *et al.* [18] atlas, RA fibres for the endocardium corresponded to the RA endocardial structures only, including PM, crista terminalis, SAN; and a representation was stored for BB as a separate layer of the model. Fibres were mapped from a choice of three atlases: the first case of an *ex vivo* DTMRI dataset (DTMRI1) [20]; the average fibre atlas from seven *ex vivo* DTMRI datasets (DTMRIA) [19]; a rule-based atlas (Labarthe) [18]. These fibre atlases, together with the other DTMRI cases, are all provided as fibre field options within the pipeline.

**Figure 4.**
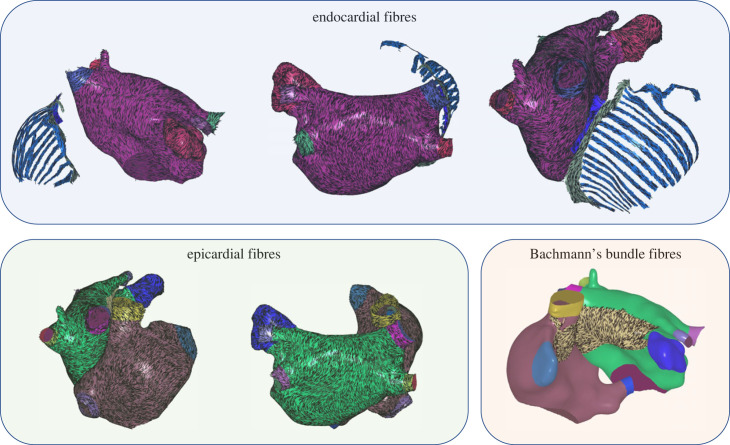
Atrial fibres for endocardial surfaces or associated structures, epicardial surfaces and Bachmann’s bundle. This example is for a case with pulmonary vein tissue.

For bilayer models, LA endocardial, LA epicardial fibres and RA epicardial fibres were each mapped from the chosen distribution. RA endocardial fibres for the PM, crista terminalis and SAN were mapped from the Labarthe fibre atlas across models. This was also the case for including BB fibres. This atlas was chosen because the Labarthe *et al.* [18] atlas captures preferential fibre directions described in these structures. Since these structures were not segmented separately and individually registered in the DTMRI atlases, the fibre directions in the DTMRI atlases may contain a mix of fibres between the specialized conduction structures and epicardial surfaces. An example of the mapping is shown in figure 4 with atrial fibres assigned to the endocardial surfaces or associated structures; epicardial surfaces and BB.

**Figure 5.**
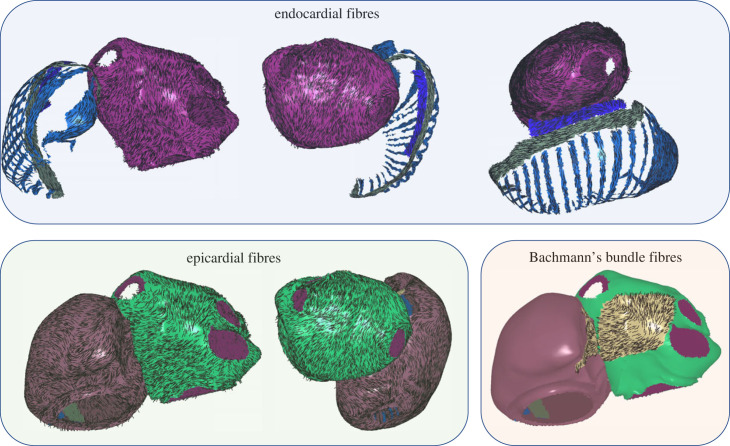
Atrial fibres for endocardial surfaces or associated structures, epicardial surfaces and Bachmann’s bundle. This example is for a case without pulmonary vein tissue.

For cases in which the PV, LAA, SVC and IVC tissue has been removed from the mesh, UACs are calculated as described in §2.3, and fibres are assigned as described above. An example is shown in figure 5 with atrial fibres assigned to the endocardial surfaces or associated structures, epicardial surfaces and BB.

### 2.7. Including atrial fibres: volumetric models

For volumetric models, atrial fibres were assigned using a similar approach as in bilayer models, using the transmural coordinate to guide the choice of fibre assignment. Before fibres were assigned to the mesh, atrial structures were added, using a similar approach to §2.4. Specifically, for the LA of volumetric meshes, fibres for a transmural coordinate of less than 0.5 were assigned as endocardial fibres, while fibres for a transmural coordinate of greater than 0.5 were assigned as epicardial fibres. For the RA of volumetric meshes, the tissue was first separated into endocardial structures including PM and crista terminalis; BB and epicardial tissue. PM and crista terminalis were defined where *α* and *β* correspond to these regions, and where the transmural coordinate was less than 0.7. BB was defined where *α* and *β* correspond to this region, and where the transmural coordinate was greater than 0.7. All other tissue was defined as epicardial.

Volumetric fibres were then assigned as the surface fibre corresponding to the *α* and *β* values from either the choice of epicardial atlas for RA body and RAA, or using the Labarthe atlas for the endocardial structures including crista terminalis and PM, and interatrial structures including BB. This is demonstrated in figure 3*c*.

### 2.8. Including atrial fibrosis

Scalar mapping approaches use UAC to register scalar datasets across different anatomies, either from the same or different patients. This scalar mapping approach was used to assign atrial fibrosis to 100 of the CT-derived biatrial models using 100 fibrosis distributions from our previously published study [1]. These image intensity ratio distributions on LA meshes were mapped using UAC to the LA and RA of the 100 CT-derived biatrial models. Fibrosis was assigned to the RA of each model using the same image intensity ratio map that was chosen for the LA since Hopman *et al.* [7] demonstrated that the amount of LA fibrosis is highly correlated with the amount of RA fibrosis.

### 2.9. Simulations and post-processing

Simulations were run to assess differences in paced wavefront propagation through calculation of local activation time maps, and in AF wavefront patterns by calculating phase singularity maps.

Specifically, simulations were run in openCARP using the Courtemanche *et al.* [21] ionic cell model with the monodomain model for tissue propagation. The ionic conductances of the Courtemanche *et al.* cell model were modified to reproduce physiological heterogeneity between regions of the atria, following our previous publication [22]. Fibrotic remodelling was included for elements with an image intensity ratio greater than 1.2 as changes in ionic conductances and conductivity, following our previous study [1].

Paced local activation time maps were calculated by stimulating each MRI model at the RSPV boundary nodes, and identifying the time the transmembrane potential reached −10 mV at each node. Phase singularity density maps were calculated from 15 s AF simulations, in which AF was automatically initiated by seeding four spiral wave re-entries [23]. Transmembrane potential signals were post-processed to calculate phase and phase singularity density maps, as we previously described [22].

## 3. Results

### 3.1. Cohorts of models constructed from MRI data, electroanatomical mapping data or CT data

**Figure 6.**
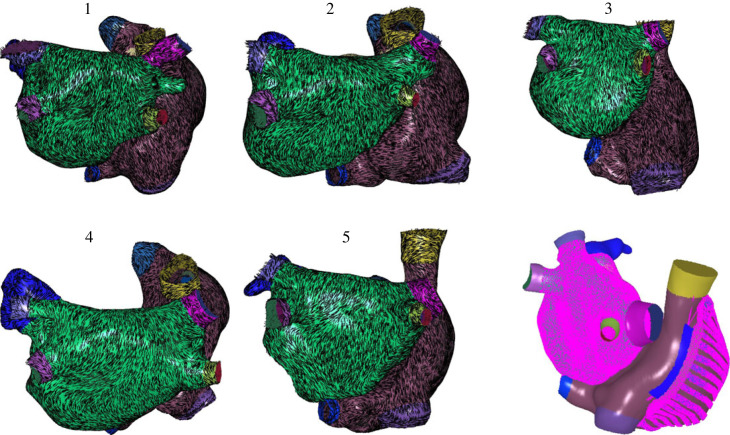
A cohort of five MRI models with fibres. The final panel shows the locations of line connections (in purple) that couple the different surfaces in the bilayer model representation.

Figure 6 shows five biatrial bilayer models constructed from MRI data, with interatrial connections included at the septum, BB and the CS. Once the meshes were clipped at the PV, SVC, IVC, CS and valves, and landmarks were selected, the process was fully automated.

**Figure 7.**
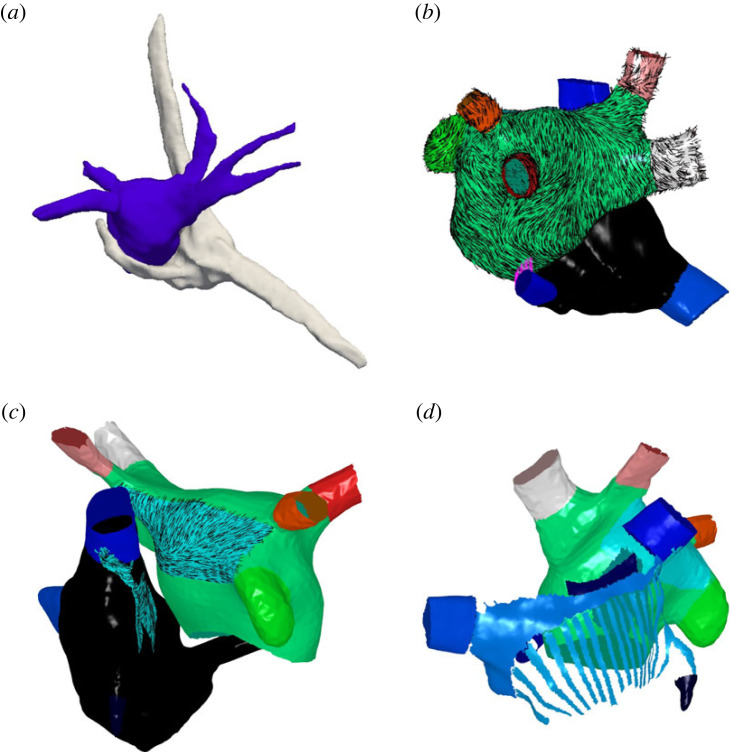
Biatrial bilayer model constructed from an electroanatomical map. (*a*) Example LA (blue) and RA (grey) meshes extracted from the Abbott system. (*b*–*d*) Atrial structures and fibres constructed from the anatomy shown in (*a*) for the following regions: (*b*) LA epicardial fibres, (*c*) BB fibres, (*d*) crista terminalis and PM regions.

Figure 7 shows an example bilayer model for a biatrial anatomy constructed from an EAM.

We also constructed 1000 biatrial bilayer models and volumetric models from a CT-derived statistical shape model. This provides a large cohort of biatrial models with anatomical structures and fibres, available as both bilayer and volumetric representations incorporating the range of healthy anatomical variability.

### 3.2. Choice of fibre field affects simulated activation patterns and atrial fibrillation dynamics

**Figure 8.**
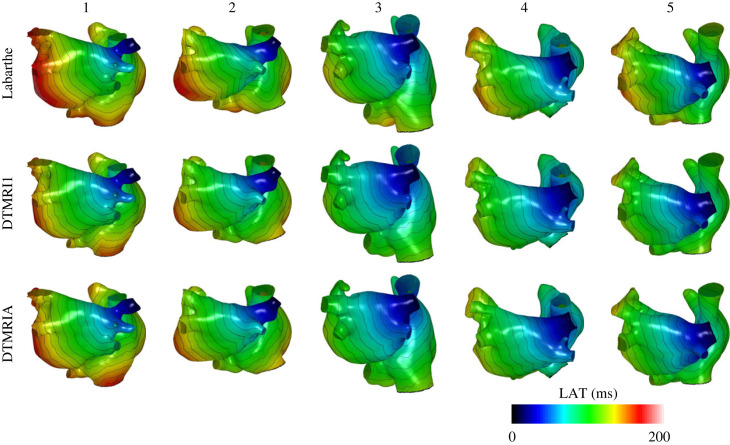
Local activation time maps across five anatomies (columns) with a choice of three fibre maps (rows).

Local activation time maps varied between anatomies and between fibre fields, as shown in figure 8. This variation was bigger between anatomies than between fibre fields, with the range of total activation times for varying fibre field and fixed anatomy being less than 12 ms, while the range of total activation times across anatomies was 45 ms (total activation times for anatomy 1: 172.5–183.7 ms, anatomy 2: 167.5–172.5 ms, anatomy 3: 139.6–142.5 ms, anatomy 4: 129.9–144.2 ms, anatomy 5: 142.6–151.3 ms).

**Figure 9.**
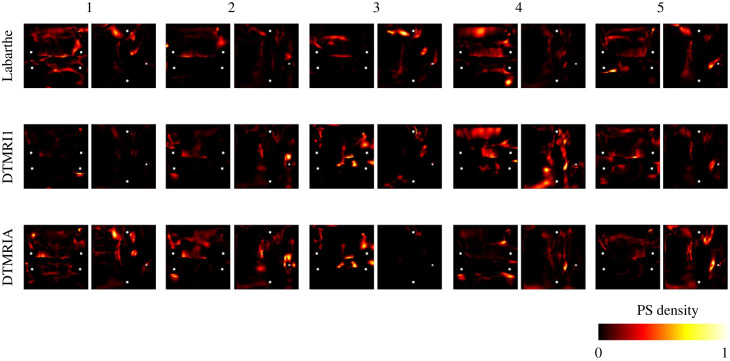
Phase singularity density maps across five anatomies (columns) with a choice of three fibre maps (rows). The colour bar is normalized phase singularity density from 0 in black through red, orange and yellow to 1 in white.

AF wavefront dynamics showed a greater sensitivity to the choice of anatomy and fibre field than the case of paced activation, as quantified through phase singularity density maps for models without fibrosis, shown in figure 9. The correlation between phase singularity density maps for DTMRI1 compared to DTMRIA is higher than between the Labarthe fibre field and the DTMRI fields (correlation between DTMRI1 and DTMRIA: 0.62 ± 0.21; DTMRI1 versus Labarthe: 0.33 ± 0.08; DTMRIA versus Labarthe: 0.32 ± 0.12, each listed as mean and standard deviation).

### 3.3. Choice of bilayer or volumetric model construction affects atrial fibrillation dynamics

**Figure 10.**
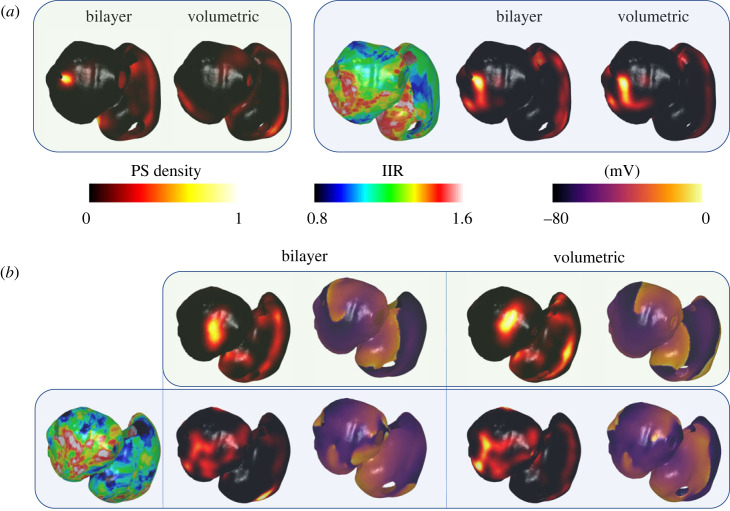
The choice of bilayer or volumetric model construction affects AF dynamics, but this effect is reduced for cases with fibrotic remodelling. (*a*) An example in which the bilayer and volumetric phase singularity density maps are different for the baseline model (first box in green). When fibrotic remodelling is added in the second box indicated by the blue background panel, the AF wavefront patterns are much more similar between the bilayer and volumetric models, resulting in similar phase singularity density maps. (*b*) A second example where the baseline models in the top row (green box) are more similar in the bilayer and volumetric models. When fibrotic remodelling is added in the bottom row (blue box), the AF wavefront patterns are again similar, with similar phase singularity density maps. IIR, image intensity ratio; PS, phase singularity.

Typically, AF patterns quickly diverged between bilayer and volumetric models resulting in visually different activities, with different phase singularity density maps. An example is shown in figure 10*a*. Phase singularity density maps for 1000 bilayer models were compared to phase singularity density maps for their equivalent volumetric representation, demonstrating a low mean correlation coefficient of 0.27 ± 0.19 for the LA, and a mean correlation coefficient of 0.41 ± 0.14 for the RA (given as mean ± standard deviation).

Table 1 shows mean and standard deviation correlation coefficients for single chamber and biatrial simulations calculated between bilayer and volumetric representations. These results are displayed as both correlation coefficients (calculated using *corr2* in Matlab) and structural similarity indices (calculated using *ssim* in Matlab). The top three rows show these results for LA only, RA only and biatrial models, with results presented for LA and RA phase singularity density maps separately.

**Table 1.** Correlation and structural similarity indices comparing bilayer and volumetric models for different model set-ups. Results are shown as mean ± standard deviation. The *corr2* function in Matlab was used to calculate the correlation coefficient, and *ssim* was used to calculate the structural similarity index.

model comparison	sample size	LA		RA	
		corr2	ssim	corr2	ssim
LA bilayer versus vol	100	0.24 ± 0.20	0.52 ± 0.09		
RA bilayer versus vol	100			0.17 ± 0.15	0.44 ± 0.09
biatrial bilayer versus vol	1000	0.27 ± 0.19	0.38 ± 0.10	0.41 ± 0.14	0.27 ± 0.06
biatrial bilayer versus vol LGE all	100	0.52 ± 0.20	0.47 ± 0.10	0.36 ± 0.18	0.41 ± 0.13
biatrial bilayer versus vol LGE (Utah1)	9	0.38 ± 0.24	0.40 ± 0.05	0.43 ± 0.12	0.34 ± 0.07
biatrial bilayer versus vol LGE (Utah2)	24	0.53 ± 0.19	0.45 ± 0.10	0.33 ± 0.15	0.31 ± 0.07
biatrial bilayer versus vol LGE (Utah3)	28	0.55 ± 0.17	0.48 ± 0.10	0.36 ± 0.17	0.40 ± 0.10
biatrial bilayer versus vol LGE (Utah4)	39	0.52 ± 0.22	0.49 ± 0.09	0.35 ± 0.22	0.51 ± 0.12

### 3.4. Effect of model type on atrial fibrillation dynamics is reduced for cases with fibrotic remodelling

Adding fibrotic remodelling to the bilayer and volumetric models typically stabilized the re-entries, making the wavefront propagation patterns visually more similar. Examples are shown in figure 10 for two examples with and without fibrotic remodelling. In both examples (figure 10*a*,*b*), the AF wavefront patterns for cases with fibrotic remodelling are similar between the bilayer and volumetric models, resulting in similar phase singularity density maps. For cases with fibrotic remodelling, the mean correlation coefficient for the LA was higher 0.52 ± 0.20, with a similar RA mean correlation coefficient of 0.36 ± 0.18.

Table 1 shows that in the case of fibrotic remodelling, the mean correlation coefficients for biatrial simulations calculated between bilayer and volumetric representations is typically increased compared to cases without any remodelling. To investigate the effects of fibrotic remodelling on these relationships, correlations and structural similarity indices were calculated for models with different degrees of fibrosis quantified by Utah grade as follows: Utah 1 (less than 10% surface area classified as fibrosis), Utah 2 (10–20% fibrosis), Utah 3 (20–30% fibrosis) and Utah 4 (greater than 30% fibrosis), thresholding fibrosis at an image intensity ratio of 1.2 [24]. Breaking down these results by Utah grade to represent different degrees of fibrotic remodelling (increasing from Utah 1 (less than 10% surface area is fibrosis) to Utah 4 (greater than 30%)), the correlation is lower for the LA for Utah 1 (0.38 ± 0.24) than for Utah 2 and above (e.g. for Utah 2: 0.53 ± 0.19).

The average number of phase singularities in the LA was similar for the bilayer and volumetric models with and without fibrosis (baseline bilayer: 1.46 ± 0.57, baseline volumetric: 1.81 ± 0.72, fibrosis model bilayer: 1.27 ± 0.52, fibrosis model volumetric: 1.08 ± 0.55, averaged across all fibrosis stages). By contrast, adding fibrotic remodelling decreased the number of phase singularities in the RA for bilayer and volumetric models (baseline bilayer: 3.92 ± 0.71, baseline volumetric: 3.39 ± 0.93, fibrosis model bilayer: 1.24 ± 0.46, fibrosis model volumetric: 1.11 ± 0.54).

LA surface area was in the range 76.1−111.3 cm^2^ (mean and standard deviation: 93.4 ± 5.9 cm^2^), while RA surface area was in the range 87.4−136.7 cm^2^ (mean and standard deviation: 109.4 ± 8.1 cm^2^). Considering the relationship between PS number and chamber surface area across 1000 cases, there was a weak positive linear relationship between RA surface area and mean PS number in the RA for bilayer models (slope 0.04, *R*^2^ 0.25). There was no relationship between RA surface area and mean PS number in the RA for volumetric models (*R*^2^ 0.06), or between LA surface area and mean PS number in the LA for bilayer or volumetric models (*R*^2^ < 0.12).

## 4. Discussion

### 4.1. Main findings

We have presented an open-source pipeline for generating atrial models at scale from imaging or EAM data. The methodology works for anatomies that include or exclude atrial structures outside of the atrial body (for example, pulmonary veins). The pipeline calculates a set of universal atrial coordinates that are used to add atrial structures to the mesh, to add connections between the atria, and to add fibres from a choice of atlas fibre fields. Models can be constructed in either bilayer or volumetric format. We applied the multi-modality pipeline to generate 1000 biatrial bilayer and volumetric models from a CT-derived set of anatomies, five biatrial bilayer meshes from MRI, and EAM meshes. We demonstrated that simulated activated pattern during regular pacing depends on anatomy and fibre field. We simulated fibrillation across the 1000 bilayer and volumetric models and found that fibrillation dynamics are sensitive to the choice of model representation. Adding fibrotic remodelling to the simulations stabilized the re-entries and reduced the impact of model choice on fibrillatory patterns. As such, we have shown that the representation of thickness appears to be secondary for informing fibrillatory wavefront patterns. Our methodology enables both patient-specific mechanistic studies and large *in silico* trials of atrial arrhythmia treatment approaches. These tools are available in an open-source interactive application to provide a user-friendly workflow.

### 4.2. Choice of fibre field

The pipeline includes seven different DT MRI fibre fields for the endocardium and epicardium of the left and right atria, as well as an average DT MRI fibre field [19], and a rule-based fibre field from Labarthe *et al.* [18]. The choice of fibre field has a small effect on local activation time fields for paced data, similar to the findings of He *et al.* [25], but it has a large effect on fibrillatory dynamics. These findings extend our previous study in which we performed LA only or RA only simulations and found small differences in local activation time maps [19]. In our current study, we find larger differences in root mean squared error because differences accumulate in biatrial models. In our previous study, we found that DTMRI fibre field 1 resulted in the most similar AF wavefront patterns to the other fibre fields, motivating our choice in this study to use DTMRI dataset 1 and the average dataset. Similarly, we found PS density maps for DTMRI1 and DTMRIa were more highly correlated to each other than to PS density maps with the Labarthe fibre atlas. In the case that EAM data are available with pacing from multiple directions, electrical anisotropy may be estimated [26,27] and these directions could be used as the fibre directions.

Previously presented methodologies for incorporating fibre directions in anatomical meshes include Piersanti *et al.* [8] who use a Laplace–Dirichlet rule-based method for the atria and ventricles. Azzolin *et al.* [9] developed the AugmentA pipeline for generating atrial models with fibres, which they demonstrated on 29 LA datasets. AugmentA uses a statistical shape model to generate a RA anatomy for a LA anatomy input meaning the pipeline has the advantage that it can produce biatrial models for LA only input meshes. We build on their approach to test our pipeline across a wide range of biatrial CT (1000 instances), MRI and EAM anatomies, demonstrating that our methodology is robust to anatomical variability. Aspects of these pipelines could be combined to enable *in silico* trials at scale.

### 4.3. Choice of model type: volumetric versus bilayer

We demonstrated that fibrillatory dynamics are sensitive to model representation, including the choice of whether a bilayer surface model, or volumetric model is used. In general, fibrillatory dynamics quickly diverged between the two representations, leading to different phase singularity maps. Adding fibrotic remodelling anchored and stabilized re-entries, and consequently reduced the dependence of the phase singularity map on model representation (see figure 10 and table 1). This suggests that once a model has been personalized to fibrosis information from imaging data, or electrophysiology measurements, the choice of whether a volumetric or bilayer representation is used has less of an effect on model predictions.

Our study also highlights the importance of personalizing conduction velocity and electrophysiological properties for informing model predictions. Once heterogeneity in activation and repolarization properties are incorporated in the model, the effects of fibres and wall thickness on AF dynamics are greatly reduced. As such, recent efforts to personalize models to LGE-MRI data [1] and EAM data [9,28] are imperative.

It should be noted that our simulations are for homogeneous thickness, and modelling variations in thickness may impact activation speed. Hansen *et al.* [29] used high resolution 3D LGE-MRI together with optical mapping to map intramural re-entry anchored to fibrosis-insulated atrial bundles in the right atria showing the importance of wall thickness and fibrosis information in arrhythmia mechanisms. Future developments in clinical imaging and mapping systems may enable these data to be integrated in computational models. However, currently it is challenging to determine wall thickness from clinical MRI or EAM data, and so a bilayer model may be an appropriate model in this case since it is computationally efficient and allows modelling of endocardial–epicardial dissociation [18].

Finally, the type of mesh used, and the resolution of the mesh may have an impact as large as true three-dimensional effects. Future studies should investigate the effects of mesh resolution [30] and heterogeneous wall thickness on arrhythmia dynamics. In addition, small spatial shifts in PS locations will decrease the correlation coefficient even if this shift is not clinically relevant. Alternative metrics could be considered to measure the difference in the centroid location of islands of high PS density, comparing this to ablation lesion size or to perform this analysis on a regional basis.

### 4.4. Modelling at scale for *in silico* trials

Our pipeline enables the construction of large virtual patient cohorts and personalized model prediction on clinical timescales by overcoming previous limitations including the requirements of significant time and expertise from trained users, significant computation time, and lack of reproducibility. We have outlined the steps involved in our pipeline, the software requirements, time taken for the different steps and areas of future improvement in table 2. As we move towards an ecosystem for digital twins in healthcare [31], open-source pipelines become increasingly more important. Rodero *et al.* [32] provide a comprehensive review of cardiac *in silico* trials highlighting that the number of cases included in current digital twin trials is typically small, with only a small number of trials including more than 50 patients. This limits the applicability of *in silico* trial results to the wider human population. Providing a pipeline, such as the one presented here, for constructing models at scale, and making these models available, should increase the size and impact of future *in silico* trials.

**Table 2.** Modelling pipeline steps, software requirements, time requirements and areas for improvement.

	model pipeline	input	source	output	software used	manual input time	computational requirements	expertise required	user friendly	future improvements
0	imaging dataset	volumetric meshes of the atria (vtk)	cohort of CT-derived statistical shape models: https://zenodo.org/records/4506930	n.a.	—	<5 min, download	0	low	yes	—
0	imaging dataset	MRI images and labels for the atria (nii)	LGE-MRI images from atrial segmentation challenge: https://www.cardiacatlas.org/atriaseg2018-challenge/	n.a.	—	<5 min, download	0	low	yes	—
1	segmentation (if starting from imaging data)	image file (nii, nrrd, DICOM)	0 imaging dataset	surface meshes of left and right atria (stl)	3D slicer (https://www.slicer.org/)	<1 h, segment images	0	high	yes—video provided for training	fully automatic segmentation: improve accuracy and speed, remove high expertise requirement
2	clipping	closed surface mesh of atria (stl)	1 segmentation	open surface mesh of atria (stl)	paraview (https://www.paraview.org/)	<10 min, make clips	0	medium	yes—video provided for training	automated clipping: improve consistency and speed, remove medium expertise requirement
3	Landmark selection	open surface mesh of atria (stl)	2 clipping	landmark coordinate files (txt)	custom Python scripts for point selection (PyVista)	<10 min, select points	0	high	yes—video provided for training	automated landmark selection: improve consistency and speed, remove high expertise requirement
4	meshing	open surface mesh of atria (stl)	2 clipping	simulation grade mesh of atria (carp)	meshtool (https://bitbucket.org/aneic/meshtool/src/master/)	<5 min, update paths	5 min—laptop	medium	yes—bash script provided to run	—
5	UAC Calculation (regions, fibres, simulation ready mesh)	simulation grade mesh (carp), Landmark coordinate files (txt)	3 landmarks, 4 meshing	simulation mesh with atrial regions and fibres (carp)	custom Python scripts, meshtool, openCARP (https://opencarp.org/)	<5 min, update paths	20 min—laptop	medium	yes—bash script provided to run	—
6	simulations	simulation mesh with atrial regions and fibres (carp), initial conditions, parameter file (par)	5 UAC calculation	spatio-temporal transmembrane potential data (igb)	openCARP (https://opencarp.org/)	<5 min, update paths	few hours—desktop or HPC	high	yes—online tutorials for training	—

Note: Start at 2 clipping if already provided with segmentation Mask, which saves ca 1 h of user time'; 5 min on a laptop; 20 min on a laptop; few hours on a desktop or HPC.

### 4.5. Limitations

This current pipeline starts from a segmentation and is not a full end-to-end methodology. Our pipeline requires the user to process anatomical shells to ensure that LA meshes are open at the PVs and MV, and that RA meshes are open at the vena cava and TV. An additional manual step is the selection of landmark points. To assign the boundaries between the PVs and LA body, the appendages and atrial body, and the vena cava and atrial body, manually chosen threshold values were used. A future development to standardize this could adaptively select this threshold so that a certain percentage area of the LA tissue forms the PV regions, or to ensure a standard average PV length. A limitation is that the pipeline assumes the presence of four PV orifices. The approach could be generalized to remove PV boundary conditions in the same way that it was modified to remove the requirement of PV and appendage tissue in the model. If data are available in the future, atlases could also be constructed for fibre fields with different numbers of PV.

### 4.6. Conclusion

We have presented an open-source pipeline for generating atrial models at scale from imaging or EAM data. The methodology enables both patient-specific mechanistic studies and large *in silico* trials of atrial arrhythmia treatment approaches. We used the methodology to demonstrate that personalization of electrophysiological properties had a greater impact on model predictions than the impact of model type (bilayer or volumetric).

## Data Availability

Codes for constructing biatrial bilayer and volumetric models are available together with examples and detailed instructions on zenodo: https://zenodo.org/records/10139306 [33]. CT-derived statistical shape four chamber anatomies are available to download from zenodo: https://zenodo.org/records/4506930 [34]. LGE-MRI fibrosis distributions for left atrial models are also available to download on zenodo: https://zenodo.org/records/5801337 [35]. OpenCARP solver is available open source for electrophysiology simulations: https://opencarp.org/.
